# Body CT examinations in oncologic patients: the impact of subspecialty radiology on radiation exposure in the clinical practice. A quality care study

**DOI:** 10.1007/s11547-024-01790-2

**Published:** 2024-02-11

**Authors:** Stefania Rizzo, Luca Bellesi, Andrea D’Ermo, Luca Bonomo, Oriana D’Ecclesiis, Francesco Magoga, Stefano Presilla, Arturo Spanò, Veronica Minzolini, Francesca Lo Piccolo, Jurgen Heinkel, Ermidio Rezzonico, Maria Del Grande, Matteo Merli, Filippo Del Grande

**Affiliations:** 1 Clinic of Radiology, Imaging Institute of Southern Switzerland (IIMSI), Ente Ospedaliero Cantonale (EOC), Via Tesserete 46, 6900 Lugano, Switzerland; 2 Faculty of Biomedical Sciences, Università Della Svizzera Italiana (USI), Via G. Buffi 13, 6904 Lugano, Switzerland; 3 Service of Medical Physics, Maging Institute of Southern Switzerland (IIMSI), Ente Ospedaliero Cantonale (EOC), Bellinzona, Switzerland; 4 Service of Process Organization and Information, EOC, Support Area, Via Lugano 4D, 6500 Bellinzona, Switzerland; 5 Department of Experimental Oncology, IEO, European Institute of Oncology IRCCS, Milan, Italy; 6 Oncology Institute of Southern Switzerland, EOC, Bellinzona, Switzerland

**Keywords:** Radiology subspecialty, Oncology, CT dose index, Dose length product, Objective image quality

## Abstract

**Purposes:**

The primary objective of this retrospective study was to assess whether the CT dose delivered to oncologic patients was different in a subspecialty radiology department, compared to a general radiology department. The secondary explorative objective was to assess whether the objective image quality of CT examinations was different in the two settings.

**Materials and methods:**

Chest and abdomen CT scans performed for oncologic indications were selected from a general radiology department and a subspecialty radiology department. By using a radiation dose management platform, we extracted and compared CT dose index (CTDI_vol_) and dose length product (DLP) both for each phase and for the entire CT exams. For objective image quality evaluation, we calculated the signal-to-noise ratio (SNR) and the contrast-to-noise ratio (CNR) at the level of the liver and of the aorta. A P-value < 0.05 was considered significant.

**Results:**

A total of 7098 CT examinations were included. CTDI_vol_ was evaluated in 12,804 phases; DLP in 10,713 phases and in 6714 examinations. The CTDI_vol_ and DLP overall were significantly lower in the subspecialty radiology department compared to the general radiology department CTDI median (IQR) 5.19 (3.91–7.00) and 5.51 (4.17–7.72), DLP median and IQR of 490.0 (342.4–710.6) and 503.4 (359.9–728.8), *p* < 0.001 and *p* = 0.01, respectively. The objective image quality showed no significant difference in the general and subspecialty radiology departments, with median and IQR of 4.03 (2.82–5.51) and 3.84 (3.09–4.94) for SNR_Liv_ (*p* = 0.58); 4.81 (2.70–7.62) and 4.34 (3.05–6.25) for SNR_Ao_ (*p* = 0.30); 0.83 (0.20–1.89) and 1.00 (0.35–1.57) for CNR_Liv_ (*p* = 0.99); 2.23 (0.09–3.83) and 1.01 (0.15–2.84) for CNR_Ao_ (*p* = 0.24) with SNR_Liv_ (*p* = 0.58), SNR_Ao_ (*p* = 0.30), CNR_Liv_ (*p* = 0.99) and CNR_Ao_ (*p* = 0.24).

**Conclusion:**

In a subspecialty radiology department, CT protocols are optimized compared to a general radiology department leading to lower doses to oncologic patients without significant objective image quality degradation.

## Introduction

Several radiology departments are changing their organization system from general to subspecialty radiology, because of the recognized importance of dedicated expertise in imaging interpretation and in multidisciplinary discussions [1–3]. It has been demonstrated that the second opinion and radiological reports provided by subspecialized radiologists are more effective, clear and appropriate than those provided by general radiologists [4, 5]. Furthermore, subspecialized radiologists’ interpretations lead to a better integration of the imaging reports into the clinical management of the patients, which help the transition from volume-based to value-based imaging care [6].

In clinical practice, radiologists must select the optimal CT imaging protocol to provide the required information with the lowest radiation dose. The imaging protocol selection may vary according to several factors such as the degree of detail of clinical indications, clinical information (e.g., age, weight, availability of previous imaging exams), mode of access, patient preferences, radiologist preference, radiology experience and other factors. In fact, marked variations can be seen in imaging protocols from one institution to another, within the same institution and between different radiologists, especially when the protocol is left to the choice of the radiologist alone [4].

In oncologic imaging, patients undergo multiple computed tomography (CT) examinations from staging to response assessment, follow-up and prognostication [7, 8], and are therefore prone to considerable radiation exposure, the cumulative dose exposure being possibly correlated with the risk of future cancers, especially in children and young adults [9, 10]. For this reason, previous and existing initiatives by scientific societies, such as the American College of Radiologists [11, 12] and the European Society of Radiology, support the adoption of dose management systems to ensure the justification and optimization of radiological procedures and store information on patient exposure for analysis and quality assurance [13].

We hypothesized that radiologists dedicated to oncologic imaging, aware of the imaging characteristics of tumors, are able to select the most appropriate CT imaging protocol compared to general radiologists, taking into account the need to keep the radiation dose as low as reasonably achievable [14].

In 2019, four radiology departments in the same geographical area were merged into one Imaging Institute and the radiologists were divided into subspecialty sections, according to their training, experience and preference. The reason for a specific oncologic imaging group relied on the presence of a dedicated oncologic institute in the same hospital, and in recognition of some particular characteristics in the evaluation of oncologic patients, such as the evaluation of response according to specific criteria (including response evaluation imaging criteria for solid tumors (RECIST), i-(immune)-RECIST, etc.), as well as to the increasing number of specific responses/progression patterns relative to target therapies or complications related to immunotherapies. This organizational change gave us the unique opportunity to compare the CT doses delivered to oncologic patients in the two organizational settings.

Therefore, the primary objective of this study was to assess whether the CT dose delivered to oncologic patients by chest and abdomen CT is different in a subspecialty radiology department, compared to a general radiology department. The secondary explorative objective was to assess whether the objective image quality of CT examinations was different in the two organizational settings.

## Methods

This retrospective study, relying on merged anonymized data, was considered a quality care control study by our Ethics Committee and did not fall under the Swiss law of human research. As such, the need for specific approval and informed consent was waived.

### CT examination selection

A business intelligence and visualization tool (Microsoft Power BI Desktop) extracted chest and abdomen CT scans performed for oncologic indications over a 12-month period when the organization was a general radiology department (01.01.2018–31.12.2018), and over a 12-month period when the organization was a subspecialty radiology department (01.01.2022–31.12.2022). The CT scans were randomly performed on 6 CT scanners available at our institution that were not changed during the two study periods (Somatom Definition Flash, Siemens Healthineers, Erlangen, Germany; 4 Somatom Definition Edge, Siemens Healthineers, Erlangen, Germany; 1 Brilliance ICT, Philips Healthcare, Eindhoven, Netherlands).

Exclusion criteria were: CT scans performed to study vascular pathologies (such as aortic aneurysms and pulmonary embolism); examinations present in the local picture archiving and communication system but performed at other institutions; and examinations including metallic prostheses. Further exclusion criterion for the whole exam DLP was the presence of a cerebral acquisition in the same exam.

### Dose report data extraction

By using a commercially available dose management platform (Radimetrics, Bayer HealthCare, Leverkusen, Germany), we extracted the radiation output for each CT phase acquisition (pre-contrast, arterial, venous and delayed phases), recorded as CT dose index (CTDI_vol_, measured in mGy), and as dose length product (DLP, measured in mGy x cm), and for the entire single- or multiphase exam, recorded as exam DLP. Since CTDI_vol_ is a measure of one slice, whereas DLP is a measure of a series including lots of slices, the number of records for each CT examination will be higher for CTDI_vol_ than for DLP. CTDI_vol_ and DLP from CT localizers and from series of few images, such as bolus triggering, were furtherly excluded.

### Objective image quality evaluation

**Fig. 1:**
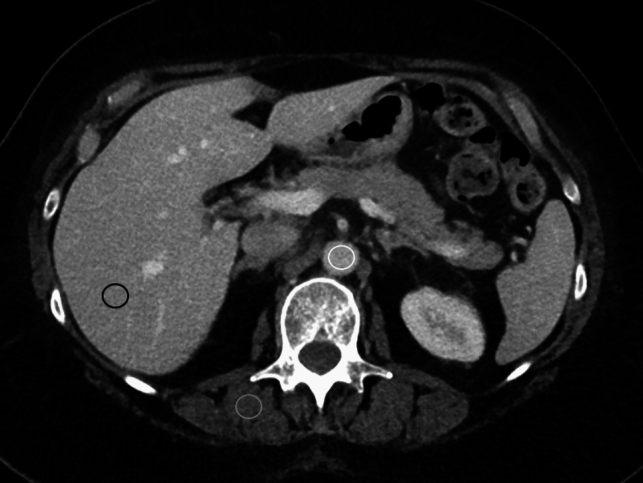
Axial CT image at the level of the first lumbar vertebra, on which regions of interest were circled within the abdominal aorta (white circle), the right liver lobe (black circle) and the paraspinal right muscles (gray circle) to evaluate objective image quality

Group of CT scans (*n* = 100) was randomly selected from the general radiology ones (*n* = 50) and from the subspecialty radiology ones (*n* = 50). The CT datasets were selected by choosing the first 4 CT exams for each month of the period selected, and the first 5 for the months of March and July (randomly chosen because of the 31 days in these months). The images were anonymized, divided into two groups and used for the objective image quality assessment. Three circular regions of interest (ROIs) measuring 15 mm^2^ were drawn by a 3rd year resident in radiology [LB], supervised by a radiologist with 19 years of experience in oncologic imaging [SR] on a single CT slice at the level of the first lumbar vertebra within the following structures: the abdominal aorta, without touching the lumen walls, to cover at least two-thirds of its lumen; the right lobe of the liver, covering a homogeneous area; and the paraspinal right muscles (Fig. 1). Mean and standard deviation (image noise) of density, measured by Hounsfield units (HU), were recorded and then used to calculate the signal-to-noise ratio (SNR) and the contrast-to-noise ratio (CNR) at the level of the liver and of the aorta, according to the following formulas [15]:

1. SNR_liv_ = HU_liv_/SD_liv_, where HU_liv_ is the mean attenuation value and SD_liv_ is the standard deviation in the liver ROI.
2. SNR_ao_ = HU_ao_/SD_ao_, where HU_ao_ is the mean attenuation value and SD_ao_ is the standard deviation in the aorta ROI.
3. CNR_liv_ = (HU_liv_-HU_m_)/√(SD^2^_liv_ + SD^2^_m_), where HU_m_ is the mean attenuation value and SD_m_ is the standard deviation in the muscle ROI
4. CNR_ao_ = (HU_ao_-HU_m_)/√(SD^2^_ao_ + SD^2^_m_)

### Statistical analysis

Kolmogorov–Smirnov test, Shapiro test and QQplot were used to test the normality of CTDI_vol_, DLP, SNR and CNR distributions. For descriptors not normally distributed, the nonparametric Wilcoxon–Mann–Whitney (WMW) test was used. Data were summarized through median and interquartile range (IQR). Quantile regression was used to assess whether radiation dose in each phase and in the entire exam were associated with general radiology and subspecialty radiology.

For all analyses, a two-tailed p-value < 0.05 was considered statistically significant. The statistical analyses were performed with R software, version 4.2.3.

## Results

According to inclusion and exclusion criteria, 7098 CT exams were included in this study (Table 1): 3073 from the general radiology department and 4025 from the subspecialty radiology department.

**Table 1:** Summary of the CT exams, phase CTDIvol, phase DLP and exam DLP included from the general radiology department and from the subspecialty radiology department

	Total (*n*)	General radiology (*n*)	Subspecialty radiology (*n*)
CT exams	7098	3073	4025
Phase CTDI_vol_	12,804	5090	7714
Phase DLP	10,713	4176	6537
Exam DLP	6714	3003	3711

The analysis of CTDI_vol_ was performed on a cohort of 12,804 phases of acquisition (*n* = 5090 acquired in the general radiology department; *n* = 7714 in the subspecialty radiology department); the analysis of DLP was performed on a cohort of 10,713 phases of acquisition (*n* = 4176 acquired in the general radiology department; *n* = 6537 in the subspecialty radiology department). The DLP of each entire exam was evaluated in 6714 CT exams (*n* = 3003 acquired in the general radiology department; *n* = 3711 in the subspecialty radiology department).

**Fig. 2:**
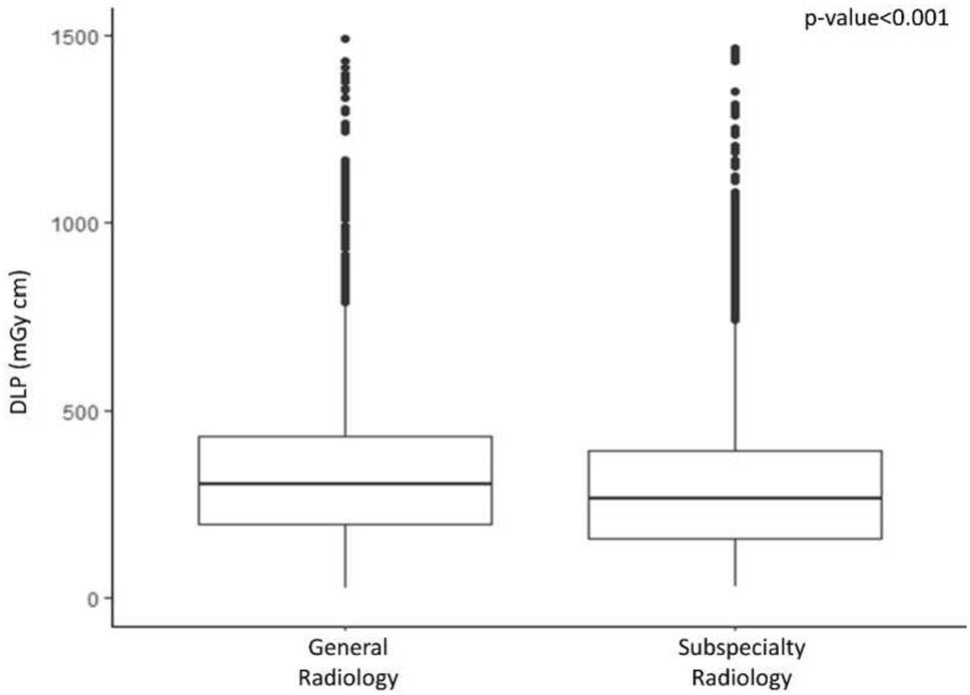
Overall DLP in CT scans acquired in oncologic patients in a general radiology department and in a subspecialty radiology department

As shown in Fig. 2, the analysis dedicated to the overall DLP showed values significantly (*p* < 0.001) lower in CT scans performed in the subspecialty radiology department as compared to the general radiology department, with median and IQR of 265.0 (160.2–393.1) and 304.2 (197.2–434.8), respectively.

**Fig. 3:**
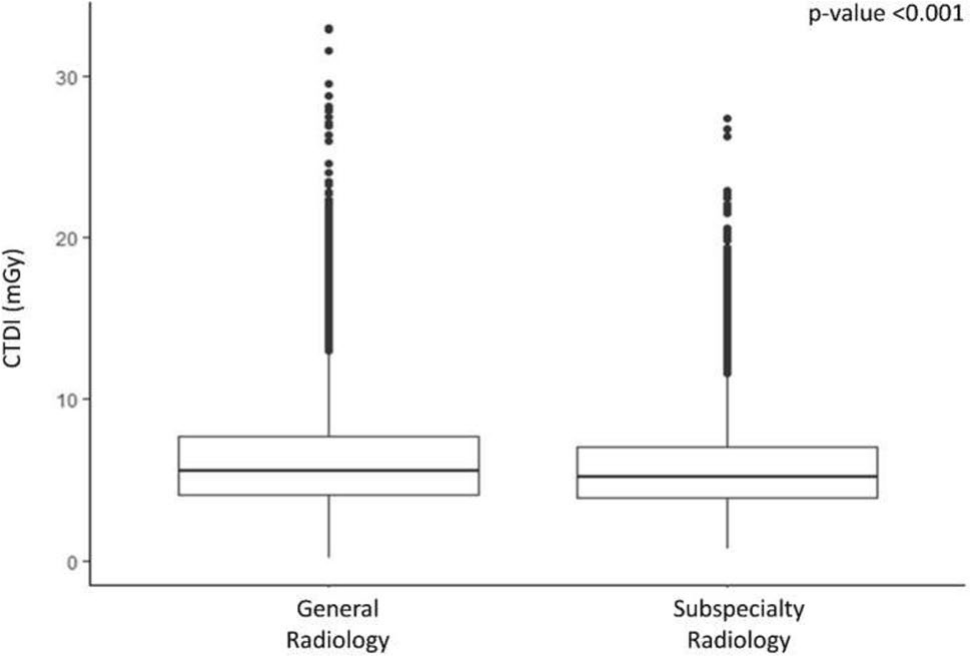
Overall CTDI_vol_ in CT scans acquired in oncologic patients in a general radiology department and in a subspecialty radiology department

**Fig. 4:**
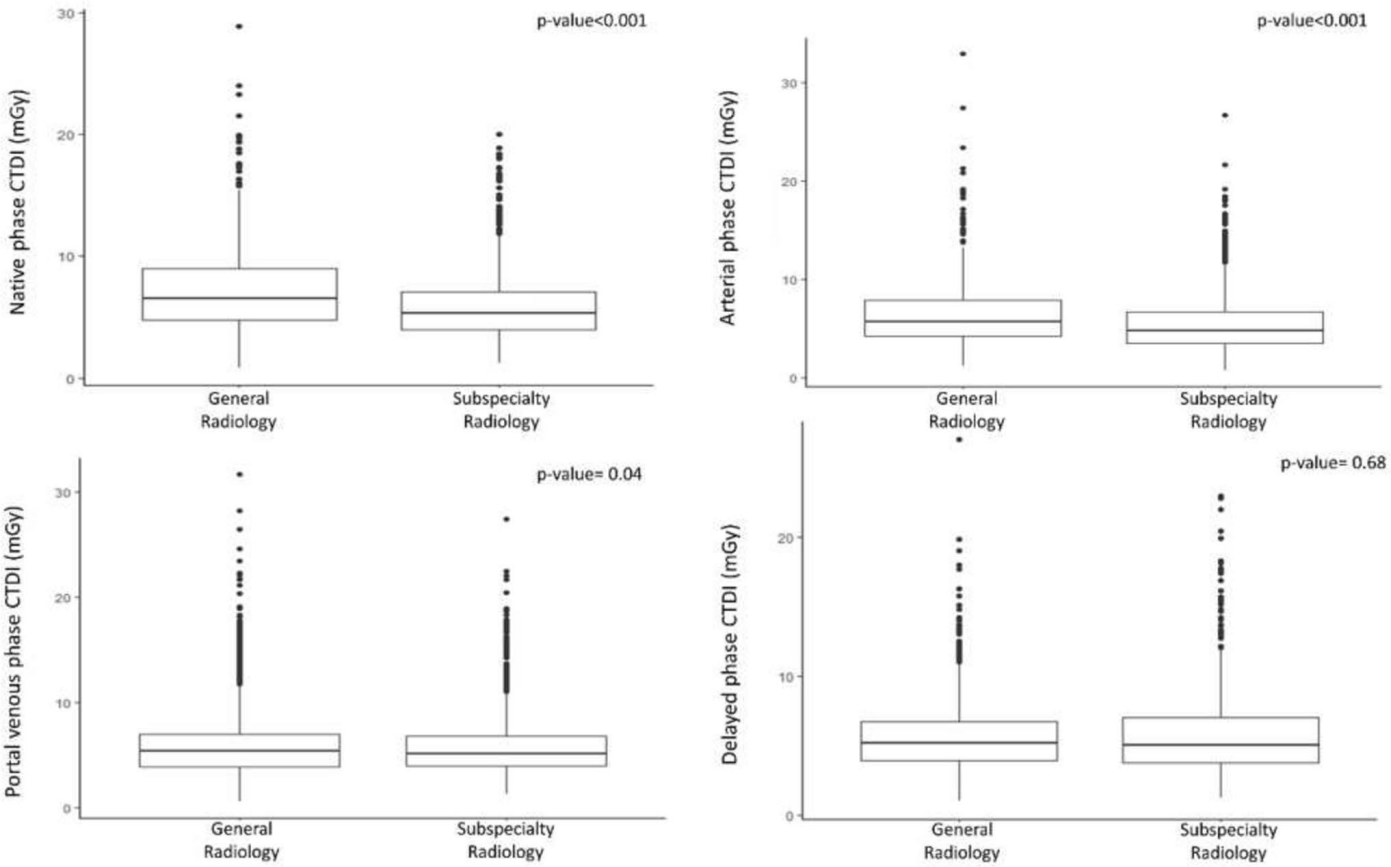
Difference of CTDI_vol_ in the pre-contrast, arterial, portal venous and delayed phase acquisitions of CT scans in oncologic patients in the general radiology department and in the subspecialty radiology department

The overall CTDI_vol_ was significantly (*p*-value < 0.001) lower in CT scans performed in the subspecialty radiology department as compared to the general radiology department [median (IQR) 5.19 (3.91–7.00) and 5.51 (4.17–7.72), respectively (Fig. 3)]. A further analysis dedicated to the single acquisition phases confirmed significantly lower values of CTDI_vol_ in the pre-contrast, arterial and portal venous phases in CT scans performed in the subspecialty radiology department, as compared to the general radiology department (Fig. 4).

As shown in Table 2, the multivariate quantile regression performed on 12,804 CTDI_vol_ records, adjusted by phase acquisition, confirmed the results of the univariate analysis, showing significantly lower values of CTDI_vol_ delivered by CT scans in the subspecialty radiology department compared to the general radiology department (*p*-value < 0.001). The same analysis, performed on all the scans according to the acquisition phase, showed a significantly higher CTDI_vol_ for pre-contrast acquisitions compared to portal venous phase acquisitions (*p* < 0.001).

**Table 2:** Quantile regression of CTDI_vol_ (Number of observation: 12,804)

	*β*	SE	*p*-value
Department			
Subspecialty versus general radiology	− 0.37	0.04	**< 0.001**
Phase			
Arterial versus portal venous	− 0.09	0.06	0.13
Pre-contrast versus portal venous	0.49	**0.07**	**< 0.001**
Delayed versus portal venous	− 0.08	0.05	0.12

Bold *p*-values (< 0.001 and < 0.001), indicate “significant *p*-values”

*SE* Standard error

**Fig. 5:**
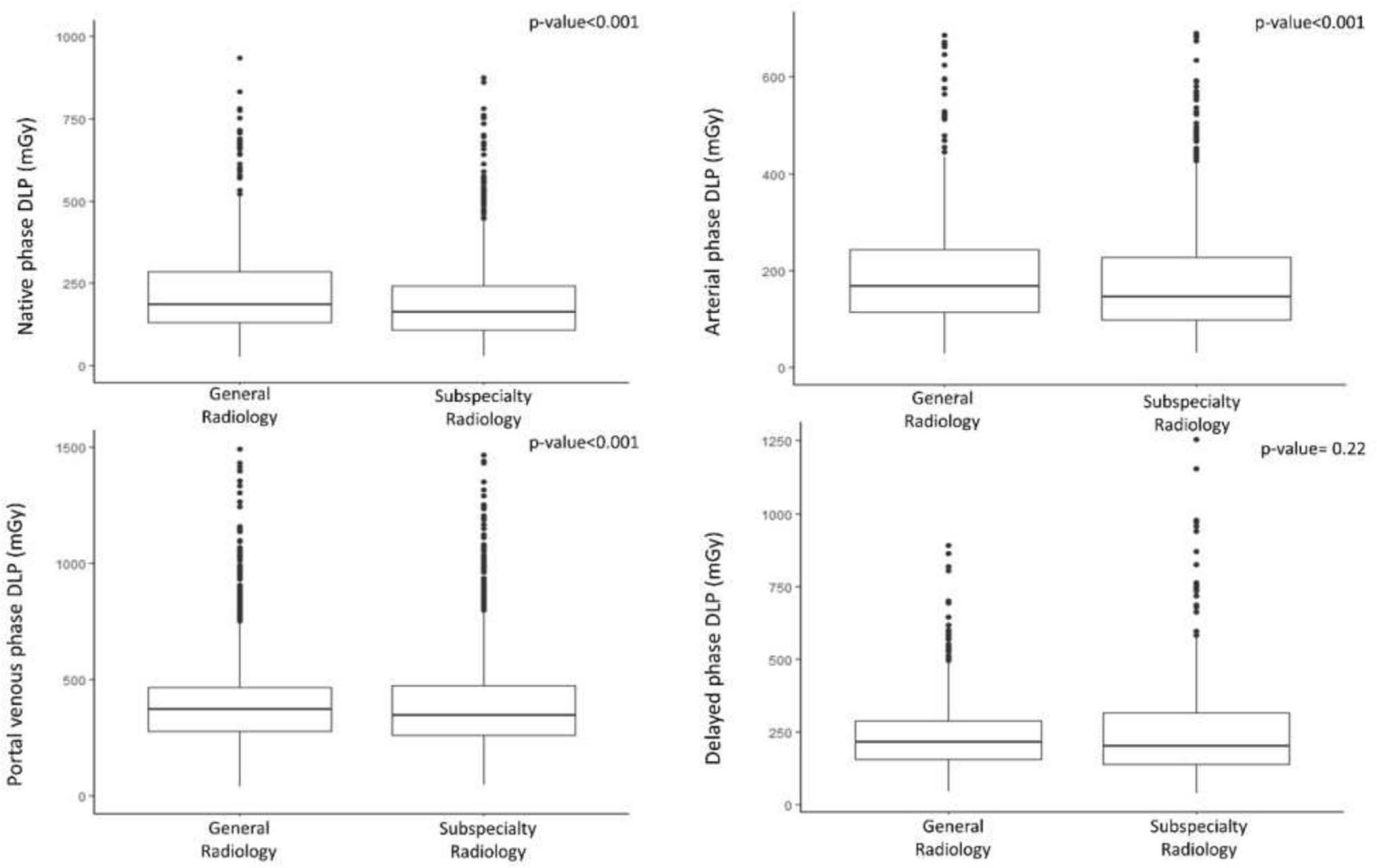
Difference of DLP in the pre-contrast, arterial, portal venous and delayed phase acquisitions of CT scans in oncologic patients acquired in a general radiology department and in a subspecialty radiology department

A further analysis dedicated to the single acquisition phases confirmed significantly lower values of DLP in the pre-contrast, arterial and portal venous phases in the subspecialty radiology department, as compared to the general radiology department (Fig. 5).

The multivariate quantile regression performed on 10,713 DLP records, adjusted by the phase acquisition, confirmed the significantly lower values of DLP delivered by CT scans in the subspecialty radiology department compared to the general radiology department (*p*-value < 0.001 Table 3).

**Table 3:** Quantile regression of DLP (number of observations: 10,713)

	*β*	SE	*p*-value
Department			
Subspecialty versus general radiology	− 21.2	3.01	< 0.001
Phase			
Arterial versus portal venous	− 201.1	3.65	< 0.001
Pre-contrast versus portal venous	− 185.5	3.97	< 0.001
Delayed versus portal venous	− 150.9	4.57	< 0.001

*SE* Standard error

**Fig. 6:**
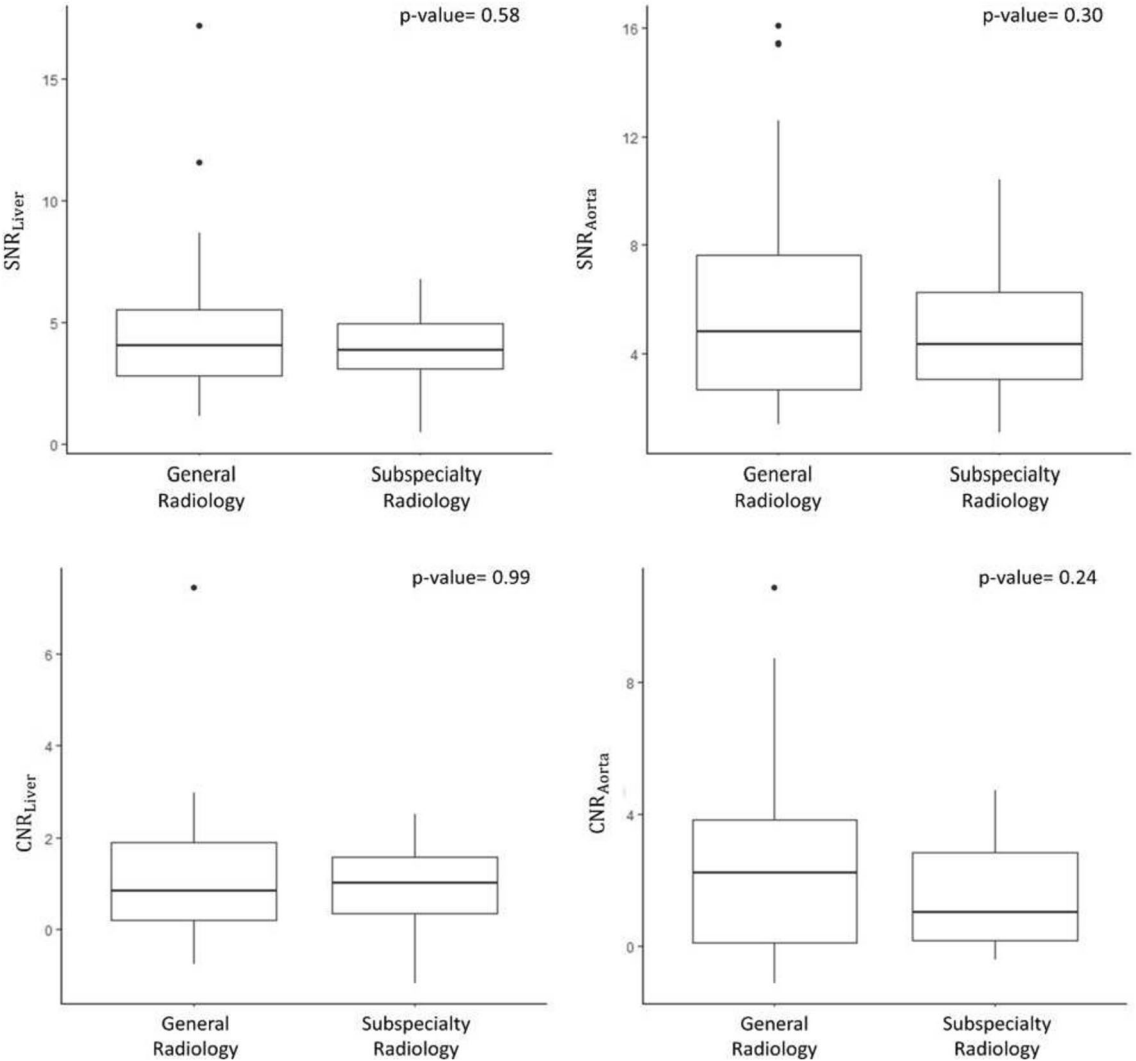
Box plots of signal-to-noise ratio (SNR) and contrast-to-noise ratio (CNR) at the level of the liver and of the aorta in the CT scans acquired in the general radiology department and in the subspecialty radiology department

The exploratory analysis dedicated to the objective image quality showed no significant difference in SNR_Liv_ (*p*-value = 0.58) and SNR_Ao_ (*p*-value = 0.30) of CT scans acquired in the general and subspecialty radiology departments, with median and IQR of 4.03 (2.82–5.51) and 3.84 (3.09–4.94) for SNR_Liv_, and of 4.81 (2.70–7.62) and 4.34 (3.05–6.25) for SNR_Ao_, respectively (Fig. 6). Similarly, there was no significant difference in CNR_Liv_ (*p*-value = 0.99) and CNR_Ao_ (*p*-value = 0.24) of CT scans acquired in the general and subspecialty radiology departments, with median and IQR of 0.83 (0.20–1.89) and 1.00 (0.35–1.57) for CNR_Liv_ and of 2.23 (0.09–3.83) and 1.01 (0.15–2.84) for CNR_Ao_, respectively (Fig. 6).

## Discussion

In our study, which included 7098 chest and abdomen CT examinations, we demonstrated that after the introduction of a subspecialty based radiology department, the CT dose for patients with oncologic indications was significantly lower compared to a general radiology department, without impairment of objective image quality.

Current CT scanners produce radiation dose sheets as a separate series in each examination. Although format and content vary between manufacturers and scanner models, all include at least the CTDI_vol_ and DLP. CTDI_vol_ indicates the average radiation output per slice, depends on the type of scanner and acquisition parameters and is independent of patient and scan length [16, 17]. DLP is the product of the CTDI_vol_ and scan length, and thus reflects the total amount of radiation to which the patient is exposed [16]. CTDI_vol_ and DLP may be helpful in assisting radiologists to comply with regulatory requirements, as well as to monitor the CT doses over time [16–20].

Some of the available current strategies to optimize radiation exposure include the use of size-dependent protocols, the use of automated exposure control, a reduction of the number of phases and a reduction of duplicated coverage [21–23]. In this study, the former two were kept constant in the two cohorts, because the CT machines and technologies were not replaced during the study periods, nor did they undergo significant software updates, whereas the CT protocols in the subspecialized radiology department were adapted taking into account the latter two.

As mentioned, automated exposure control was present in the general radiology as well as in the subspecialty department, and is demonstrated by the higher CTDI_vol_ of the native phase (usually focused on the upper abdomen) compared to the portal venous phase (including the chest and the abdomen), because, as known in the literature, the latter includes anatomical structures (e.g., the lungs) that need a lower radiation dose for optimal visualization [24, 25] Since the effect on radiation dose of multiphase CT examinations can be similar to multiple examinations, special attention should be paid to optimizing CT protocols in oncologic patients, e.g., by eliminating one or more phases [21, 22]. In a tertiary care medical center, Guite et al. demonstrated that over a 4-month period one-third of all abdominal phases resulted in an excess of effective radiation in many multiphase abdominal CT examinations [26, 27].

Previous studies demonstrated that CT dose reduction strategies also include the imaging skills of the radiologist [27–30]. Indeed, low expertise may lead to the choice of an unnecessary multiphase protocol, due to the fear of missing something. Furthermore, in a heterogeneous group of radiologists there can be a wide variability in protocol selection according to personal preferences and experience. In this regard, the inclusion of the DLP in radiological reports is a helpful way of paying constant attention to the radiation dose delivered by a CT scan examination.

In our study, the lower radiation dose of the subspecialized radiologists is likely because the subspecialized radiologists agreed on choosing protocols according to the clinical indication, the setting (staging, response to therapy assessment, follow-up) and the tumoral characteristics [16], in order to keep the dose as low as possible [14], possibly choosing different examinations if the indication was not appropriate [31]. For instance, at the first staging CT exam, the subspecialized radiologists agreed to acquire a protocol including three phases on the liver (one pre-contrast, one arterial and one portal venous phase), with the portal venous phase extending to the thorax and pelvis [32]. At the subsequent CT scans, they agreed to acquire just the portal venous phase, unless the tumor under evaluation was hypervascular (e.g., neuroendocrine tumors, renal clear cell carcinomas, breast cancers, hepatocellular cancers, melanomas) or lesions visible only in the delayed phase (e.g., urothelial tumors). This protocol strategy helped with an overall reduction of radiation exposure to the patients, as reflected by the lower DLP per exam in the subspecialty radiology, reflecting the overall exposure of the CT exam. However, this dose reduction was not strictly related to a lower number of phases acquired, as shown by 4176 acquisitions among the 3073 examinations performed by the general radiology staff, and 6537 acquisitions among the 4025 examinations performed by the subspecialty radiology staff. Indeed, since the collection of data was retrospective here, no single a priori action, such as the sole reduction of the number of acquisitions, was taken in order to see its effect on radiation dose. The dose reduction was therefore likely an integral result of the strategies altogether, rather than one alone.

Furthermore, by frequently acquiring only one phase (the portal venous phase), duplicated coverage was avoided. Indeed, scan length may not be given due attention in a busy clinical setting, although its effect on patient dose can be significant [19, 33]. Accordingly, Campbell et al. found that nearly 100% of chest CT examinations performed over a 2-week period at a large medical institution included additional images over the lung apices and below the lungs, which led to excessive radiation exposure that may even be double what is really necessary [34].

Surprisingly, aside from a lower number of delayed phases acquired, we did not find any significant difference in CT dose of the delayed phase. This result may be explained by the abovementioned adherence of the CT protocols to the clinical indication. Indeed, the delayed phase was not acquired by default in each exam, but only according to specific indications, such as in the presence of urothelial tumors, where relatively high doses are needed to detect small urothelial lesions, requiring a high spatial resolution and adequate signal- and contrast-to-noise ratios, and coverage may be limited from top of the kidneys to just below the bladder (mid pubic symphysis) [32].

Radiation staff education is another very efficient way to reinforce good practice in radiological institutes and to reduce the radiation dose to patients [35]. In a study aiming to evaluate the role of staff training events specifically designed for radiologists and technologists to achieve optimization of CT protocols, the authors demonstrated that staff training led to a significant reduction of the radiation dose associated with CT procedures (−  39.2% in unenhanced chest CT examinations and −  32.1% in contrast-enhanced whole-body CT examinations, respectively) [36]. In this study we could not perform a similar analysis, because the staff training on radiation dose was performed by radiologists and technicians regularly, according to local rules that recommend participating in one refresher teaching course, divided into four progressive modules, every 4 years, and the last one was completed in 2018–2022. Therefore, in the two periods under evaluation (2018 and 2022), all the people involved received the same teaching modules and the same training materials.

The objective of a CT scan is to obtain diagnostic images which can help to answer a clinical question in the most dose-efficient manner. Image noise is approximately inversely proportional to the square root of the radiation dose, meaning that the radiation dose must change in inverse proportion to the slice thickness to maintain constant image noise for varying reconstructed slice thicknesses [16]. With this in mind, in a subgroup of examinations we assessed whether the lower radiation dose demonstrated in the subspecialty radiology was associated with a lower image quality. This analysis did not show any significant difference in objective image noise, thus confirming that the CT protocols were correctly chosen to reduce the dose, without impairing the image quality.

This study has some limitations. One is that we did not evaluate the patients’ exposure through the effective dose, which is currently used to quantify the overall risk of fatal and non-fatal cancers, induced by ionizing radiation [14]. However, the ICRP 147 clearly states that effective dose is not the most appropriate quantity for making comparisons between doses for the same technique, where modality-specific dose quantities displayed on equipment (e.g., CTDI_vol_ and DLP) should be used to simplify the process and to avoid unnecessary approximations [37]. For this reason, we did compare CTDI_vol_ and DLP. Another limitation is that we cannot exclude the possibility that other factors, not evaluated here, may have influenced the difference in radiation dose. In this regard, the main other factor that could lead to such a difference would have been a replacement or an in-depth software update of one or more CT machines. However, the CT machines were not replaced in the period selected, and we can therefore exclude this as a confounding factor. Another potential confounding factor not evaluated in this series is the distribution of examinations over the 6 CT machines that might obscure a non-uniformity of the distribution. However, the patients are sent to one site or another of our multisite hospital according to their preference, mainly due to the proximity to their home, and this did not change either in the general radiology department or in the subspecialty department; the distribution therefore corresponded to a real-life scenario. Furthermore, although we evaluated a high number of CT scans (*n* = 7098) from the same subspecialty (oncologic imaging), we cannot confidently extend our conclusions to other subspecialties, but we may hopefully encourage similar evaluations in other subspecialties. Last but not least, we did not evaluate the effects of the reduction of radiation dose [38] and we did not consider the effects of the choice of a different imaging technique, such as magnetic resonance [39], because it was beyond the scope of our objectives and deserves dedicated prospective trials.

In conclusion, chest and abdomen CT protocols are optimized in a subspecialty radiology department compared to a general radiology department, leading to a lower radiation dose to oncologic patients without significant objective image quality degradation. This result further supports the benefit for patients of a subspecialty-based organization of radiology.

## Abbreviations

ACR: American college of radiology
ALARA: As low as reasonably achievable
CNR: Contrast-to-noise ratio
CTDI_vol_: Computed tomography dose index volume
DLP: Dose length product
HU: Hounsfield unit
ROI: Region of interest
SNR: Signal-to-noise ratio
